# Sinensetin protects against periodontitis through binding to Bach1 enhancing its ubiquitination degradation and improving oxidative stress

**DOI:** 10.1038/s41368-024-00305-z

**Published:** 2024-05-11

**Authors:** Zhiyao Yuan, Junjie Li, Fuyu Xiao, Yu Wu, Zhiting Zhang, Jiahong Shi, Jun Qian, Xudong Wu, Fuhua Yan

**Affiliations:** Department of Periodontology, Nanjing Stomatological Hospital, Affiliated Hospital of Medical School, Institute of Stomatology, State Key Laboratory of Pharmaceutical Biotechnology, School of Life Sciences, Nanjing University, Nanjing, China

**Keywords:** Target validation, Ubiquitylation

## Abstract

Periodontitis is a chronic inflammatory and immune reactive disease induced by the subgingival biofilm. The therapeutic effect for susceptible patients is often unsatisfactory due to excessive inflammatory response and oxidative stress. Sinensetin (Sin) is a nature polymethoxylated flavonoid with anti-inflammatory and antioxidant activities. Our study aimed to explore the beneficial effect of Sin on periodontitis and the specific molecular mechanisms. We found that Sin attenuated oxidative stress and inflammatory levels of periodontal ligament cells (PDLCs) under inflammatory conditions. Administered Sin to rats with ligation-induced periodontitis models exhibited a protective effect against periodontitis in vivo. By molecular docking, we identified Bach1 as a strong binding target of Sin, and this binding was further verified by cellular thermal displacement assay and immunofluorescence assays. Chromatin immunoprecipitation-quantitative polymerase chain reaction results also revealed that Sin obstructed the binding of Bach1 to the HMOX1 promoter, subsequently upregulating the expression of the key antioxidant factor HO-1. Further functional experiments with Bach1 knocked down and overexpressed verified Bach1 as a key target for Sin to exert its antioxidant effects. Additionally, we demonstrated that Sin prompted the reduction of Bach1 by potentiating the ubiquitination degradation of Bach1, thereby inducing HO-1 expression and inhibiting oxidative stress. Overall, Sin could be a promising drug candidate for the treatment of periodontitis by targeting binding to Bach1.

## Introduction

Periodontitis is a chronic inflammatory and immune-reactive disease affecting periodontal supporting tissues. It is mainly caused by the imbalance between subgingival bacteria plaque and host immune defense, resulting in the destruction of periodontal tissues and even tooth loss, which is the main cause of tooth loss in adults.^1,2^ Periodontal infection leads to a large number of pathogenic bacteria and toxic products entering oral deep tissues and spreading throughout the body. It is reported that periodontitis is associated with a variety of systemic diseases, such as cardiovascular diseases, diabetes, respiratory diseases, etc., which seriously affects human health.^3^ Host immune inflammatory response and oxidative stress play important roles in the occurrence and development of periodontitis and are also important causes of systemic diseases such as diabetes and rheumatoid arthritis.^4^ Periodontal subgingival plaque biofilm triggers the host to produce a local immune inflammatory response, producing inflammatory cytokines, stimulating reactive oxygen species (ROS) production and oxidative stress damage of periodontal tissues. Studies have found that people who are susceptible to ROS are more likely to develop periodontitis than those who have a normal response to ROS.^5^ Moreover, in periodontitis, bone destruction induced by excessive osteoclast formation and activation is mediated by the host immune and inflammatory response to the microbial challenge.^6^ BTB-CNC homology 1 (Bach1), a transcriptional factor, has always been a key role in response to oxidative stress. Recent some research for Bach1 focuses on the osteoclastogenesis which is induced by Receptor activator of nuclear factor-κB ligand (RANKL). The inhibition of Bach1 attenuates RANKL-mediated osteoclastogenesis and bone destruction by diminishing of intracellular ROS signaling.^7^ Additionally, Bach1 plays an important role in the genetic risk for severe periodontitis.^8^

At present, the main clinical treatment for periodontitis is the mechanical removal of plaque biofilm through subgingival scaling and root planning (SRP). However, for some susceptible patients, the therapeutic effect is often unsatisfactory.^9^ Therefore, it is necessary to develop adjuvant therapies to control the progression of periodontitis, such as local or systemic use of antibiotics, nonsteroidal anti-inflammatory drugs, etc.^10^ However, the problem of antimicrobial resistance and the side effects of such drugs cannot be ignored, which limits their clinical applications.^11^ Immunotherapy has become a current research hotspot for periodontitis treatment. In addition to removing plaque biofilm and other local irritants, regulating the host’s excessive immune response and oxidative stress can alleviate periodontal bone resorption. Natural compounds are considered as potential therapeutic strategies for patients susceptible to periodontitis due to their regulatory effect on the host inflammatory response and the absence of side effects.^12^ Adjuvant therapy with drugs that regulate host inflammatory response will have better clinical efficacy for patients with systemic diseases such as diabetes and rheumatoid arthritis.

Sinensetin (Sin) is a polymethoxylated flavone (PMF) found in *Orthosiphon aristatus* var. *aristatus* and certain citrus fruits, which has been studied for its ability to prevent diseases.^13,14^ As many other compounds derived from citrus like nobiletin,^15^ Sin has been confirmed to have neuroprotective, anticancer, anti-obesity and anti-diabetic effects.^16,17^ The anti-inflammatory activity of Sin has been also confirmed by studies. Researchers investigated the anti-inflammatory activity of Sin by using LPS-stimulated RAW 264.7 cells. LPS produces an inflammatory response by activating the MAPK and NF-ĸB pathways involved in the degradation of inhibitor ĸB (iĸB). Sin inhibits NF-ĸB activation by inhibiting the degradation of IĸB-α, therefore inhibiting excessive inflammation.^18^ At the same time, Sin was found to dose-dependently inhibit prostaglandin E2 (PGE2) production and inhibit the expression of iNOS and COX-2. Sin was observed in AML2/DX100 cells with antioxidant activity comparable to vitamin E.^19^ It not only has a variety of beneficial pharmacological effects, but also has minimal toxicity and high selectivity for normal cells.^20^ In this context, we hypothesized that Sin may also have a beneficial effect on periodontitis.

To test this hypothesis, we evaluated the antioxidant and anti-inflammatory properties of Sin in human periodontal ligament cells (PDLCs) exposed to the inflammatory environment and in an experimental model of periodontitis. Additionally, we explored the specific molecular mechanisms by which Sin alleviates oxidative stress, highlighting its potential as a novel and promising drug candidate for the treatment of periodontitis.

## Results

### Sin attenuated oxidative stress and inflammatory levels of PDLCs under inflammatory conditions

**Fig. 1:**
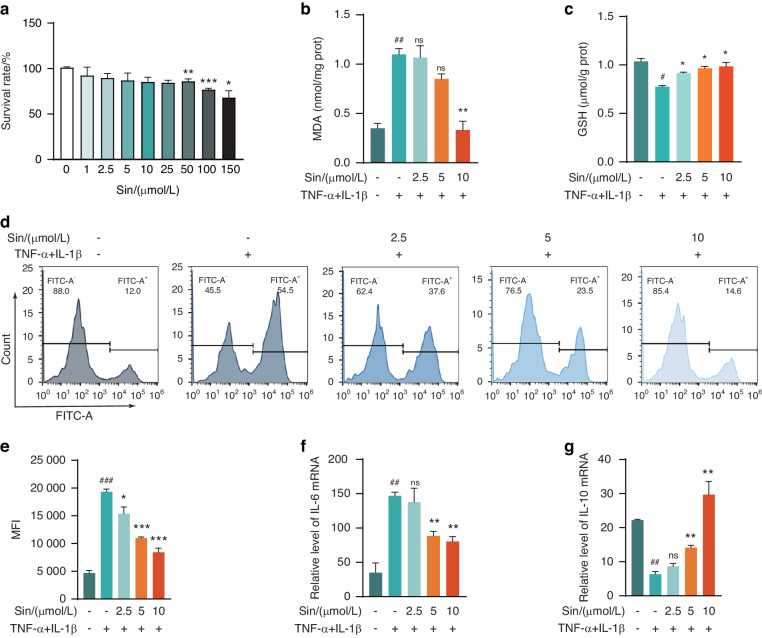
Antioxidant and anti-inflammatory properties of Sin. **a** Sin cytotoxicity tested by CCK-8 assay (*n* = 3). **b** Detection of MDA content in PDLCs (*n* = 3). **c** Detection of GSH content in PDLCs (*n* = 3). **d** Measurement of ROS production in PDLCs by Flow cytometry (*n* = 3). **e** Statistics of mean fluorescence intensity (MFI) in **d**. **f** IL6 mRNA expression in PDLCs treated with Sin at different concentrations (*n* = 3). **g** IL10 mRNA expression in PDLCs treated with Sin at different concentrations (*n* = 3). Data are presented as the mean ± SD, when compared with control group, *P < 0.05, **P < 0.01, ***P < 0.001 in (A). When compared with control group, #*P* < 0.05, ##*P* < 0.01, when compared with TNF-α + IL-1β group, *P < 0.05, **P < 0.01 in **b**, **c**, **e**–**g**

To determine the appropriate concentrations of Sin, we conducted CCK-8 assays on PDLCs to assess cell viability at various concentrations of Sin. Based on these results, we selected 2.5, 5, and 10 μmol/L as the doses for Sin treatment (Fig. 1a). To explore the impact of Sin on oxidative stress, we measured MDA, GSH levels, and intracellular ROS production and found that Sin significantly attenuated TNF-α and IL-1β-induced oxidative stress, as evidenced by decreased MDA and ROS levels and increased GSH content (Fig. 1b–e). Additionally, we observed that Sin reduced the mRNA expression of IL6 and upregulated the mRNA expression of IL10 in PDLCs exposed to inflammatory conditions, indicating its anti-inflammatory activity (Fig. 1f, g). Collectively, these results suggest that Sin exerts significantly inhibitory effects on oxidative stress and inflammatory levels of PDLCs under inflammatory conditions.

### Bach1 serves as a key target for Sin exerting antioxidant effects

**Fig. 2:**
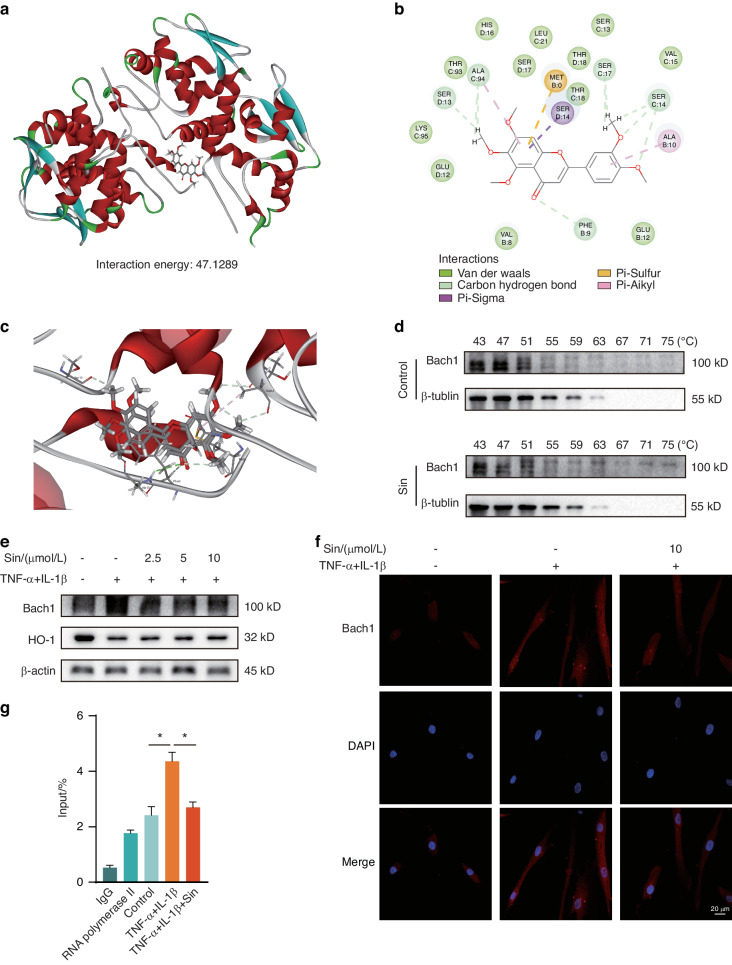
Bach1 serves as a key target for Sin to exert its antioxidant effects. **a** Molecular docking of Sin with Bach1. **b**, **c** Sites prediction of Sin combined with Bach1. **d** The result of cellular thermal displacement assay (CETSA) of Bach1’s degradation in PDLCs without/with Sin. **e** Changes in the expression of Bach1 and HO-1 at different Sin concentrations (*n* = 3). **f** Confocal image of Bach1 under inflammatory conditions and Sin-treated conditions. **g** The binding of the promoter of the HMOX1 genes with Bach1 was investigated by ChIP‒qPCR. Data are presented as the mean ± SD, *P < 0.05, **P < 0.01 in **f**, **g**

To explore the specific mechanism of Sin’s antioxidant capacity, we employed molecular docking technology to identify key targets for Sin. Through docking Sin with Bach1, we found that Sin could bind to Bach1 with a very strong interaction energy of 47.128 9 (Fig. 2a). Bach1 is a transcriptional suppressor involved in the regulation of oxidative stress response, inhibiting the expression of heme oxygenase-1 (HO-1), a key factor in antioxidant and anti-inflammatory processes.^21–23^ Additionally, we found that Sin could bind to the Phe9, Ser13, Ser14, Ser17, and Ala94 residues of Bach1 through hydrogen bonds, indicating the strong binding between Sin and Bach1 (Fig. 2b, c). We verified the intracellular binding of Bach1 to Sin using cellular thermal displacement assay experiments and found that the thermal stability of Bach1 increased in the presence of Sin compared to the control group, indicating the binding of Sin to Bach1 (Fig. 2d). Western blot (WB) results showed that Sin dose-dependently reduced intracellular Bach1 levels in the inflammatory environment while significantly increasing HO-1 levels in PDLCs (Fig. 2e and Supplementary Fig. 1a, b). Immunofluorescence confocal microscopy results also confirmed the effect of Sin on Bach1 reduction. Fluorescence intensity analysis revealed that 10 μmol/L Sin had a significant impact on Bach1 levels (Fig. 2f and Supplementary Fig. 1c). It is known that Bach1 functions as a transcription inhibitor by binding to the promoter fraction of HMOX1, thereby regulating HMOX1 gene expression. Our chromatin immunoprecipitation-quantitative polymerase chain reaction results showed a significant increase in Bach1 binding to the HMOX1 promoter region under inflammatory conditions. After treatment with Sin, the amount of Bach1 binding was significantly reduced, indicating that Sin modulated HO-1 expression under inflammatory conditions by obstructing the binding of Bach1 to the HMOX1 promoter (Fig. 2g). In summary, these findings suggest that Bach1 serves as a key target for Sin to exert its antioxidant effects.

### Bach1 participated in the antioxidant capacity of Sin under inflammatory conditions

**Fig. 3:**
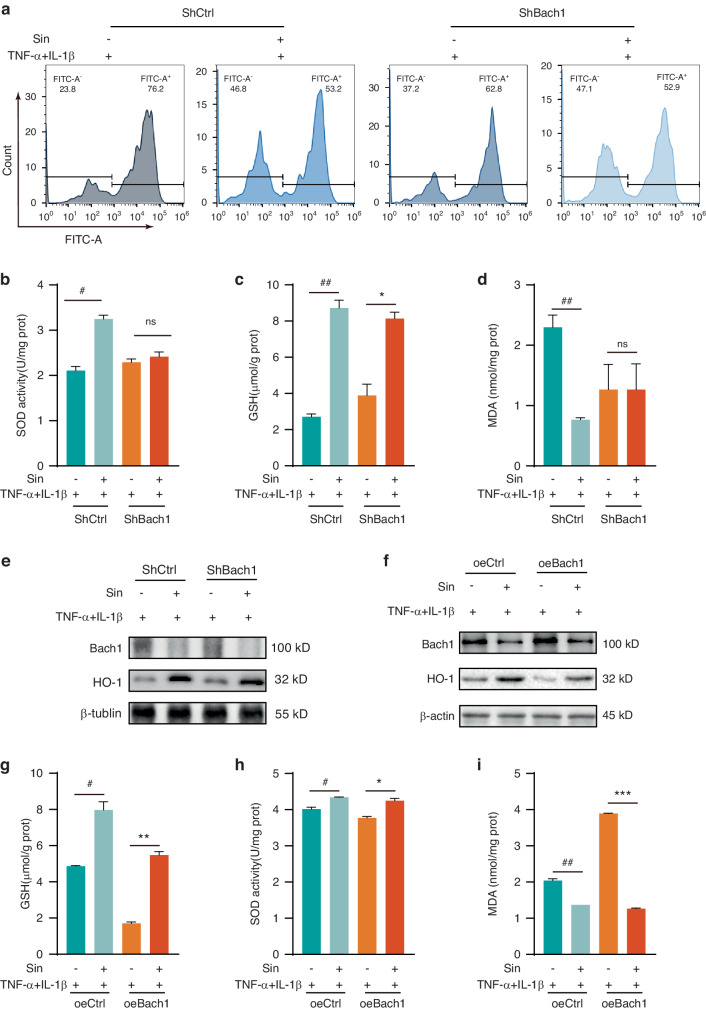
Bach1 participates in the antioxidant capacity of Sin under inflammatory conditions. **a**–**e** Changes of oxidative stress markers, including ROS formation (**a**), SOD activity (**b**), GSH content (**c**), MDA production (**d**) after knockdown of Bach1 under inflammatory conditions without/with Sin. **e** Expression of Bach1 and HO-1 after knockdown of Bach1 under inflammatory conditions without/with Sin. **f** Expression of Bach1 and HO-1 after overexpression of Bach1 under inflammatory conditions without/with Sin. **g**–**i** Changes of oxidative stress levels, including GSH production (**g**), SOD activity (**h**), MDA content (**i**) after overexpression of Bach1 under inflammatory conditions without/with Sin. Data are presented as the mean ± SD, #*P* < 0.05, ##*P* < 0.01, ###*P* < 0.001, *P < 0.05, **P < 0.01, ***P < 0.001

To further verify that Bach1 is a target of Sin’s antioxidant effect, we used lentiviral transfection to knock down and overexpress Bach1 in PDLCs (Supplementary Fig. 2f, g), and then respectively evaluated the antioxidant capacity of Sin. The results showed that knockdown of Bach1 did not significantly change the effect of Sin on ROS and GSH levels under inflammatory conditions, but caused Sin losing its ability to increase SOD and decrease MDA (Fig. 3a–d and Supplementary Fig. 2a). Compared with the ability of Sin to upregulate HO-1 in the ShCtrl group, the ability of Sin to increase HO-1 level was lowered in the ShBach1 group (Fig. 3e and Supplementary Fig. 2b, c). Therefore, knockdown of Bach1 partially weakened the antioxidant capacity of Sin.

On the contrary, after overexpression of Bach1, the effect of Sin on GSH under inflammatory conditions was more significant than the oeCtrl groups, but there was no significant change in its ability to increase SOD (Fig. 3g, h). Additionally, overexpressing Bach1 made the consumption of MDA induced by Sin under inflammatory conditions more significant than the oeCtrl groups (Fig. 3i). Moreover, the results of Western Blot showed that the effect of Sin increasing HO-1 was amplified by the overexpression of Bach1, which suggested Bach1 involves in Sin’s antioxidant effect in inflammation conditions (Fig. 3f and Supplementary Fig. 2d, e).

Overall, these results indicated that Bach1 participated in the antioxidant capacity of Sin under inflammatory conditions.

### Sin downregulated Bach1 levels through promoting the ubiquitination degradation of Bach1

**Fig. 4:**
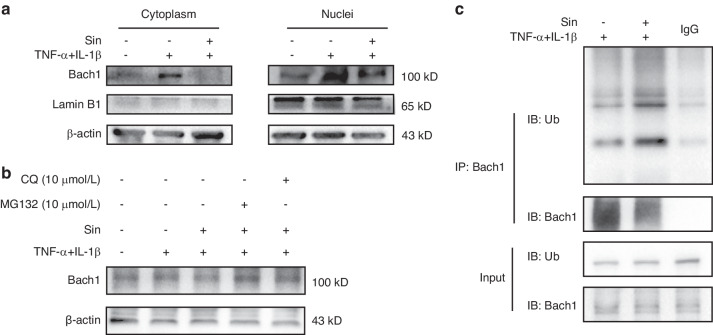
Sin promotes ubiquitinated degradation of Bach1. **a** The cytoplasmic and intranucleus levels of Bach1 under normal and inflammation conditions with/without the treatment of Sin. **b** The level of Bach1 under inflammation conditions treated with Sin and inhibitors CQ, MG132. **c** Co-IP assays of Ubiquitin (Ub) and Bach1

To gain further insight into the nuclear and cytoplasmic Bach1 content, we demonstrate that Sin reduces Bach1 levels in both the nucleus and cytoplasm (Fig. 4a). We postulated that Sin can potentiate intracellular degradation mechanisms leading to Bach1 degradation. The ubiquitin-proteasome system (UPS) and lysosomal proteolytic pathway are the two main intracellular protein degradation pathways that regulate various cellular processes, including cell cycle control, cell signaling, stress response, apoptosis, autophagy, protein expression regulation, and DNA transcription.^24–27^ We respectively added inhibitors of UPS (MG132) and lysosomal proteolytic pathway (CQ), and found that MG132 has a stronger cumulative effect on Bach1 compared to CQ, indicating that Sin mainly prompted the reduction of Bach1 by potentiating the ubiquitination degradation of Bach1 (Fig. 4b). To test this hypothesis, we performed the co-immunoprecipitation experiment with antibody of Ubiquitin (Ub) and Bach1. The results showed that in the inflammation environment Bach1 increased but did not bind to Ub at a high level. Then treatment with Sin significantly increased the binding of Bach1 to Ub (Fig. 4c). The above results signified that Sin could reduce Bach1 level through potentiating Bach1 ubiquitination degradation. Hereby, we concluded that the regulatory effect of Sin on Bach1 mainly depends on potentiating the ubiquitination degradation of Bach1, which leads to a decline of Bach1 level.

### Sin has the anti-inflammatory activity in periodontitis

**Fig. 5:**
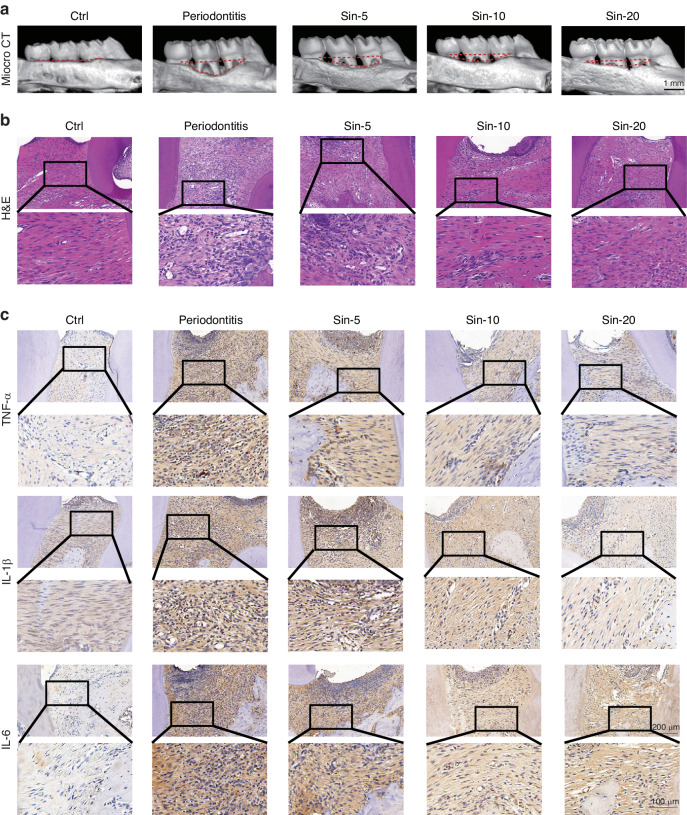
The protective effect of Sin against periodontitis in vivo. **a** Micro CT imaging of bone resorption in periodontitis in mice. **b** H&E staining of periodontium. **c** IHC staining of TNF-α, IL-1β, and IL-6 in periodontium

To further evaluate the effect of Sin against periodontitis in vivo, we administered Sin to rats with ligation-induced periodontitis models by oral gavage. As shown in images of Micro CT, alveolar bone resorption was obvious in periodontitis (PD) group induced by tooth ligation. Administration of Sin at 10 mg/kg body weight concentration (Sin-10) reduced alveolar bone resorption around the ligation region (*P* < 0.05), and 20 mg/kg Sin (Sin-20) reduced the alveolar bone loss more significantly (*P* < 0.01), indicating that Sin exhibits a protective effect against periodontitis (Fig. 5a and Supplementary Fig. 3a).

To investigate the anti-inflammatory activity of Sin, we measured the numbers of inflammatory cells using H&E staining and the levels of TNF-α, IL1-β, and IL-6 in periodontium by immunohistochemistry (IHC), respectively (Fig. 5b, c and Supplementary Fig. 3b–e). The numbers of inflammatory cells and inflammatory cytokine levels in periodontium were significantly increased in PD group, but the increase was reversed by treatment with Sin. In summary, administration of Sin (10 mg/kg) daily for three weeks effectively inhibited alveolar bone loss and inflammatory cytokine levels caused by periodontitis.

### There is a protective effect of Sin against periodontitis in vivo

**Fig. 6:**
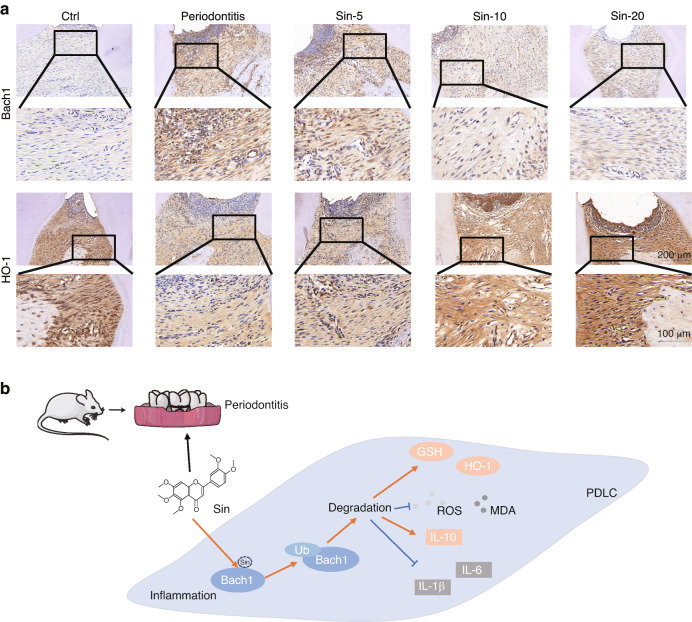
Sin protects against periodontitis by targeting Bach1. **a** IHC staining of Bach1 and HO-1 of periodontium. **b** Sin promotes the ubiquitination degradation of Bach1 by binding to it, therefore improving oxidative stress and inflammatory levels, which eventually relieves periodontitis

Moreover, we explored the mechanism of Sin targeting Bach1 in the rat periodontitis model. By IHC staining, we found that administration of Sin inhibited the level of Bach1 and elevated the expression of antioxidant factor HO-1 in periodontium, which is consistent with the results shown before (Figs. 2e and 6a and Supplementary Fig. 3f, g). Totally, Sin binds to Bach1, promoting the ubiquitination degradation of Bach1, which leads to improving the levels of oxidative stress and inflammation in periodontitis (Fig. 6b).

## Discussion

Overactivation of host immune response caused by the imbalance of periodontal subgingival plaque biofilm is an important event involved in the occurrence and development of periodontitis, which eventually leads to local or even systemic inflammation. Various natural compounds have been proved to modulate the host immune response and act as an anti-inflammatory mediator to control the development of periodontitis, therefore considered as an effective adjuvant treatment strategy. For example, resveratrol, a polyphenolic phytoalexin obtained from various plants and fruits, has a vital impact on periodontitis along with alveolar bone loss by inhibiting inflammatory responses and stimulating antioxidant defense.^12^ Resveratrol has been observed to reverse osteocyte ferroptosis induced by the diabetic periodontitis condition via regulation of the SLC7A11/GPX4 axis.^28^ Besides, genistein, a phytoestrogen from soy products, also protects against alveolar resorption and suppressed inflammatory cytokines in the mouse model of periodontitis when administered at a dose of 20 mg/kg. Additionally, plant extracts such as curcumin,^29^ proanthocyanin,^30^ and quercetin,^31^ have also exhibited protective effects against experimental periodontitis. Meanwhile Mechanistic studies suggest that flavonoids may counteract the pro-inflammatory effects produced by pathogen-associated molecular pattern (PAMP) proteins through a Toll-like receptor (TLR) response.^32^ In the present study, sinensetin, a flavonoid extracted from citrus fruits, was found to suppress alveolar bone resorption and inflammatory cytokine levels in periodontium induced by periodontitis in a novel mechanism by which Sin targets Bach1 via Bach1/OH-1 pathway.

Sin had a significant dose-dependent improvement effect on oxidative stress and inflammatory factor secretion under inflammatory conditions of PDLCs cells, as shown in Fig. 1. To explore the specific molecular mechanism, we used molecular docking to obtain the binding information of Sin and Bach1 (Fig. 2a–c). Since Bach1 is an important oxidative stress-related transcription factor that directly regulates the expression of HMOX1, this suggests that Sin plays an important role in the mechanism of oxidative stress.^7,33,34^ By knockdown and overexpression of Bach1, we found that Sin had corresponding reduction and enhancement effects on the improvement of PDLCs in inflammatory environments, which further indicated that Bach1 is an important target for Sin to improve inflammation (Fig. 3). Since Sin treatment led to a significant decrease in intracellular Bach1 content in an inflammatory environment, we found that Sin promoted its degradation mainly in the ubiquitination pathway after binding to Bach1, and ultimately reduced Bach1 level, thereby producing corresponding oxidative stress (Fig. 4).

It has been elucidated that sinensetin and its metabolites are effective regulators of the composition of the gut microflora in obese individuals.^35^ In addition, Sinensetin has also been reported to have the potential to regulate lipid metabolism by downregulating adipogenesis and related protein expression.^36^ Previously, Sin has been found to exert certain anti-inflammatory effects in a dose-dependent manner, such as regulating IκB-α and inhibiting the production of PGE2 and COX2.^18^ Our research focuses on the effect of Sin on oxidative stress. In addition to discovering the regulatory effect of Sin on oxidative stress products and related proteins, we also find the key target Bach1, where Sin works. In conclusion, our experimental results show that Sin has significant antioxidant activity and elucidates the specific mechanism, which provides new ideas for the treatment of periodontitis in the future.

HO-1 is a crucial factor involved in antioxidant and anti-inflammatory processes, activating cellular protection. Bach1 and Nrf2 competitively bind to the HO-1 promoter region and participate in the regulation of oxidative stress response.^21,37^ Inhibition of Bach1 could increase the expression of HO-1 and suppress osteoclastogenesis.^38^ Our previous study has found that long-term inflammatory stimulation increased the expression of Bach1 and oxidative stress levels in PDLCs and reduced the production of HO-1. Bach1 inhibition could reverse the oxidative damage of PDLCs caused by inflammation. Therefore, compounds that targeting Bach1 to alleviate oxidative damage and inflammatory response are thought to possess therapeutic effects on periodontitis and other inflammatory diseases.^39^ However, there is not one clinical drug targeting Bach1 and only pre-clinical research about inhibitors of Bach1 is conducted. Here, we find that Sin can strongly bind Bach1 at multiple sites, promote the degradation of Bach1, affect the regulatory effect of Bach1 on downstream oxidative stress-related factors, and ultimately achieve the effect of regulating oxidative stress. These findings indicate that Sin effectively protects against the cellular inflammatory response and bone destruction caused by periodontitis via the Bach1/HO-1-mediated signaling.

In conclusion, our study reveals the therapeutic effect and mechanism of Sin on periodontitis. Through in vivo and in vitro experiments, our exploration finds that Sin promotes the degradation of Bach1, which leads to improved levels of downstream oxidative stress. This provides a new approach to the treatment of periodontitis in the future.

## Materials and methods

### Cell culture

The PDLCs were purchased from ScienCell Research Laboratories (ScienCell, #2630). Cell culture and inflammatory stimulation have been described as previous study.^40^ PDLCs were cultured in DMEM containing 10% FBS and 1% penicillin/streptomycin. PDLCs between passages 2 and 5 were used for subsequent experiments. 10 ng/mL TNF-α and 5 ng/mL IL-1β was added to the culture medium to simulate inflammatory stimulation.

### CCK-8 assay

PDLCs were inoculated in 96-well plates and incubated with different doses of Sin for 24 h. Then, 20 μL of CCK-8 antibody-blocking peptide (Bioss) was added to each well. The plate was then placed in an incubator for 4 h. Finally, the absorbance at 450 nm was measured using a microplate reader. Cell viability was calculated based on the absorbance value of each well.

### SOD, MDA, and GSH assay

PDLCs were seeded in 6-well plates and cells were collected separately 24 h later. After cells resuspended in PBS containing PMSF, ultrasonic disruption was performed using an ultrasonic disruptor. Cells were sonicated and quantified using the BCA Protein Detection Kit. Superoxide dismutase (SOD) detection kit (WST-1 method; Nanjing Jiancheng Bioengineering Co., Ltd.), cell malondialdehyde (MDA) detection kit (colorimetry; Nanjing Jiancheng Bioengineering Co., Ltd.) and the Reduced Glutathione (GSH) Assay Kit (Microplate method; Nanjing Jiancheng Bioengineering Co., Ltd.) were used to measure SOD activity, MDA and GSH levels in PDLCs according to the manufacturer’s instructions. Antioxidants and oxidation products were estimated to explore the effect of Sin on cellular redox responses.^41^

### ROS detection

The intracellular ROS levels in PDLCs from different groups were determined using a Reactive Oxygen Species Assay Kit (Beyotime). The fluorescent probe DCFH-DA diluted with serum-free medium was added to the medium at a final concentration of 10 μmol/L. The cells were incubated for 20 min at 37 °C and washed twice with PBS. The cells were harvested, resuspended in serum-free medium, and transferred into flow tubes for centrifugation and washing. Finally, cell suspension samples were detected by flow cytometry to analyze the percentage of ROS-positive cells.

### Lentivirus transfection

The lentiviral vectors containing shRNA targeting Bach1 and overexpression of Bach1 were purchased from GeneChem. Co. (Shanghai, China). The lentiviral vector and transfection procedure have been described as the previous study.^39^

### Cellular thermal shift assay

PDLCs were inoculated in 100 mm dishes and cultured to 90% confluency. Cells were then treated with 10 μmol/L Sin or an equal volume of DMSO for 2 h. Cells were harvested and washed twice with PBS. 500 μL PBS (containing PMSF) was added to resuspend the cells, and they were immersed in liquid nitrogen and freeze-thawed 3 times. Centrifugation at 12 000 g for 10 minutes was then performed to remove the pellet. The supernatant was divided into 9 PCR tubes, each containing 20 μL, and placed in a PCR machine to receive different temperature stimuli for thermal denaturation. Centrifugation at 20 000×g for 20 min was then performed to remove the precipitate, and the supernatant was analysed by western blotting.

### Molecular docking

The molecular structure of Sin was depicted by ChemOffice and then opened in Discovery Studio (DS) to optimize the compound structure. The crystal structure of Bach1 was downloaded from the PDB database and optimized using DS. Based on the protein structure, precise molecular docking with Sin was carried out. The corresponding three-dimensional and two-dimensional interaction images were generated, and the interaction energy was sorted and analyzed.

### Western blotting

Total proteins were extracted using RIPA buffer containing 1 mmol/L PMSF. The proteins were separated by electrophoresis on the SDS-PAGE gel and transferred to PVDF membrane (Millipore). The membrane was blocked in 5% BSA buffer for 1 h at room temperature and then incubated overnight at 4 °C with the following primary antibodies: anti-Bach1 (1:1 000, Proteintech), anti-HO-1 (1:1 000, Proteintech), anti-GPX4 (1:1 000, CST), anti-β-tublin (1:3 000, Proteintech), and anti-β-actin (1:2 000, Affinity Biosciences). Finally, the target protein bands were visualized using ECL luminescence reagents (NCM Biotech) and the imaging system (Bio-Rad ChemiDoc^TM^ XRS + ).

### Immunofluorescence staining

PDLCs were cultured in 24-well plates, and after the cells adhered to the wells, cells were incubated with different stimulus conditions for 24 h. Then we aspirated the medium and gently shook the cells with PBS buffer. Cells are fixed in 1% paraformaldehyde for 30 min and permeabilized in PBS with 0.1% Triton X-100 for 20 min at room temperature. Then cells were incubated with 3% goat serum for 1 h to block non-specific binding sites. Fixed cells are incubated overnight with primary antibodies against Bach1 (1:200, Proteintech) at 4 °C, followed by 1 h at room temperature with fluorescent (FITC)-conjugated goat anti-rabbit IgG (1:1 000, Thermo Fisher Scientific). Nuclei were stained with DAPI for 15 min. All samples were imaged with Laser confocal scanning microscope (Leica TCS SP8-MP). Image J Software (NIH) was used to calculate the ratio of positive expression area to total fields of Bach1.

### Quantitative real-time polymerase chain reaction

Total RNA was isolated from PDLCs and reverse-transcribed into cDNA, followed by quantitative PCR using the BioRad CFX96 Real-Time PCR Detection System (BioRad), and threshold cycle numbers were obtained using BioRad CFX manager software. The cycling program was 1 cycle of 95 °C for 2 min, followed by 40 cycles of 95 °C for 10 s and 60 °C for 30 s. The primer sequences used in this study were as follows: IL-6 forward: ACTCACCTCTTCAGAACGAATTG, reverse: CCATCTTTGGAAGGTTCAGGTTG; IL-10 forward: GACTTTAAGGGTTACCTGGGTTG, reverse: TCACATGCGCCTTGATGTCTG. The relative amount of the IL6 or IL10 gene was normalized by the amount of β-actin and then reported as a fold change at the basal level.

### Chromatin immunoprecipitation-quantitative polymerase chain reaction

DNA and proteins in cells were cross-linked using 1% formaldehyde solution under physiological conditions. Chromatin was broken down by ultrasound treatment, and anti-Bach1 antibody or IgG antibody was added to precipitate the cross-linked complex. Precipitate of DNA fragments binding to Bach1 or IgG was then collected. De-crosslinking was performed, proteins were removed with proteases treatment, and DNA fragments were purified. DNA sequences specifically binding to Bach1 or IgG were screened by qRT-PCR. The primer sequences used are HMOX1 promoter forward: TCACAGTATTGGGAAAGGACTG, reverse: GTGGACTCCTTAGAACTCGGG; Gapdh promoter forward: CACAGTCCAGTCCTGGGAAC, reverse: TAGTAGCCGGGCCCTACTTT. During data analysis, the IgG binding amount was subtracted from the Bach1 binding amount to obtain the relative amount.

### Co-immunoprecipitation

PDLCs were collected and lysed to extract total protein. A small portion of the harvested cell lysate was used for western blotting, and the remainder was incubated overnight with 4 μg Bach1 antibody at 4 °C and precipitated with protein A/G magnetic beads (Thermo Fisher Scientific) for 1 hour at room temperature. The magnetic beads were washed four times with washing buffer (PBS buffer containing 0.05% Tween 20) on a magnetic scaffold. The immunoprecipitated protein was then boiled in loading buffer for 10 min. Finally, immunoprecipitated proteins were detected by western blotting.

### Experimental periodontitis model in vivo

The animal experiment protocol was reviewed and approved by the Laboratory Animal Welfare and Ethical Review of Nanjing University (No: IACUC-2003053). The animal feeding procedures and the establishment of experimental periodontitis models followed methods that have been described as our previous publications. In brief, 7-week-old male SD rats were housed in an SPF environment and adaptively fed for one week. A total of 15 rats were randomly divided into five groups:

Control group (Ctrl),

Periodontitis group (PD),

Periodontitis group treated with 5 mg/kg per day Sin (Sin-5),

Periodontitis group treated with 10 mg/kg per day Sin (Sin-10),

Periodontitis group treated with 20 mg/kg per day Sin (Sin-20).

The periodontitis model was established by ligating 4-0 silk sutures around the bilateral maxillary second molar for three weeks. Sin was dissolved in CMC-Na and administered to rats by oral gavage. After 3 weeks, all rats were sacrificed by overdose with anesthetics, and the bilateral maxillae were fixed with 4% paraformaldehyde for 48 h.

### Micro-CT and histological analysis

To evaluate the loss of the periodontal alveolar bone, the maxillae containing the ligation areas were scanned using micro-CT. Additionally, to estimate the alveolar bone loss, we measured the distance from the cementoenamel junction (CEJ) to the alveolar crest at six different points. The average value of all measurements was recorded as the final alveolar bone loss.

After the Micro-CT analysis, the maxillae samples were decalcified and then embedded in paraffin. The paraffin blocks were sliced to produce 4 μm thin sections, which were then stained with hematoxylin and eosin (H&E). Immunohistochemistry analysis was conducted using anti-TNF-α (GB11188, Servicebio), anti-IL-1β (GB11113, Servicebio), anti-IL-6 (GB11117, Servicebio), anti-IL-10 (GB12108, Servicebio), anti-Bach1 (BS72938, Bioworld), and anti-HO-1(10701-1-AP, Proteintech) antibodies, and the results of staining were quantified using ImageJ software.

### Statistical analysis

GraphPad Prism 8.0 was used to analyze the experimental data, and the experimental results were expressed as the mean ± standard deviation. Independent sample *t*-test was used to compare the differences between the two groups, and one-way analysis of variance was used to compare the differences between multiple groups. When the *P* value < 0.05, a significant difference was considered.

### Supplementary information

Supplementary Figures

## Data Availability

All raw data will be made available by the corresponding authors upon reasonable request.
