# Supplementary material for: Sinensetin protects against periodontitis through binding to Bach1 enhancing its ubiquitination degradation and improving oxidative stress
Source: Int J Oral Sci. 2024 May 11;16:38. doi: 10.1038/s41368-024-00305-z (PMC11088688; doi:10.1038/s41368-024-00305-z)

Supplementary Fig.1 (a-b) quantitative analysis of Bach1 and OH-1 in Fig 2e. (c) fluorescence intensity analysis of confocal image in Fig 2f. Data are presented as the mean ± SD, when compared with control group, #p<0.05, ##p<0.01, ###p<0.001, when compared with TNF-α+IL-1β group, *p < 0.05, **p < 0.01 in (a, b, c).

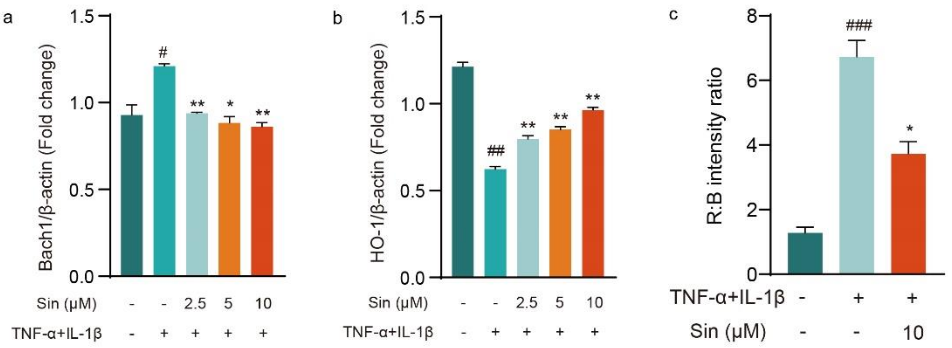

Supplementary Fig.2 (a) mean fluorescence intensity analysis in Fig 3a. (b-c) quantitative analysis of Bach1 and OH-1 in Fig 3e. (d-e) quantitative analysis of Bach1 and OH-1 in Fig 3f. (f) knockdown of Bach1. (g). overexpression of Bach1. Data are presented as the mean ± SD, #p<0.05, ##p<0.01, ###p<0.001, *p < 0.05, **p < 0.01, ***p < 0.001.

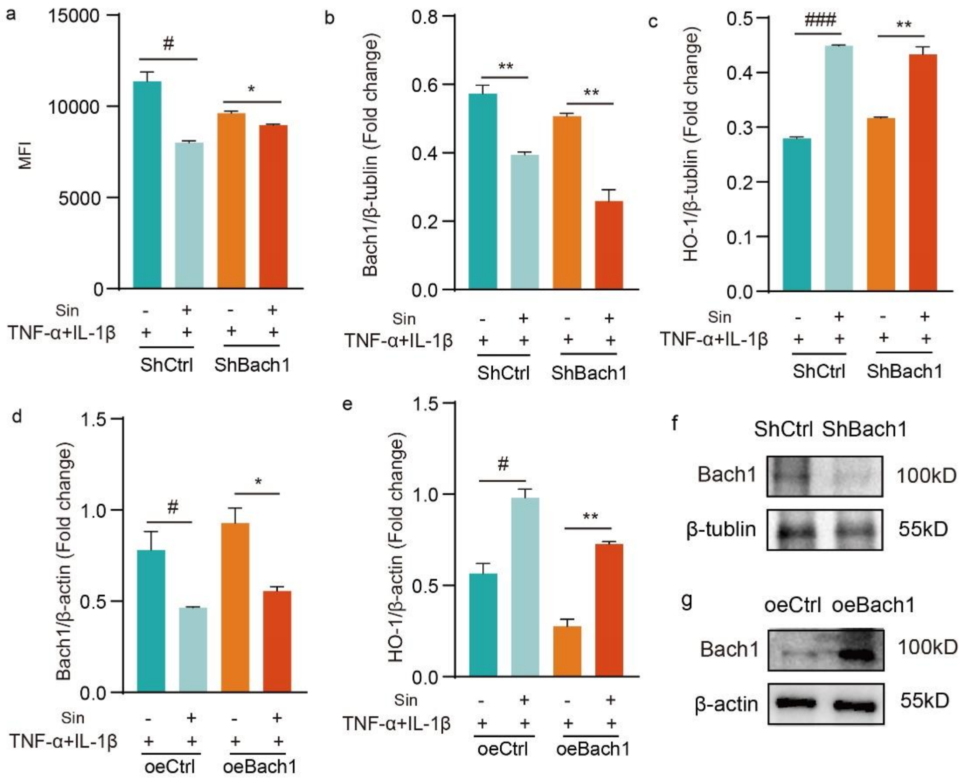

Supplementary Fig.3 (a) absorption distance of Micro CT imaging in periodontitis in mice. (b) number of immune cells in H&E staining of periodontium. (c-e) positive area percentage of TNF-α, IL-1β and IL-6 in IHC staining of periodontium. (i-g) positive percentage of Bach1 and OH-1 in IHC staining of periodontium. Data are presented as the mean ± SD, when compared with control group, #p<0.05, ##p<0.01, when compared with PD group, *p < 0.05, **p < 0.01.

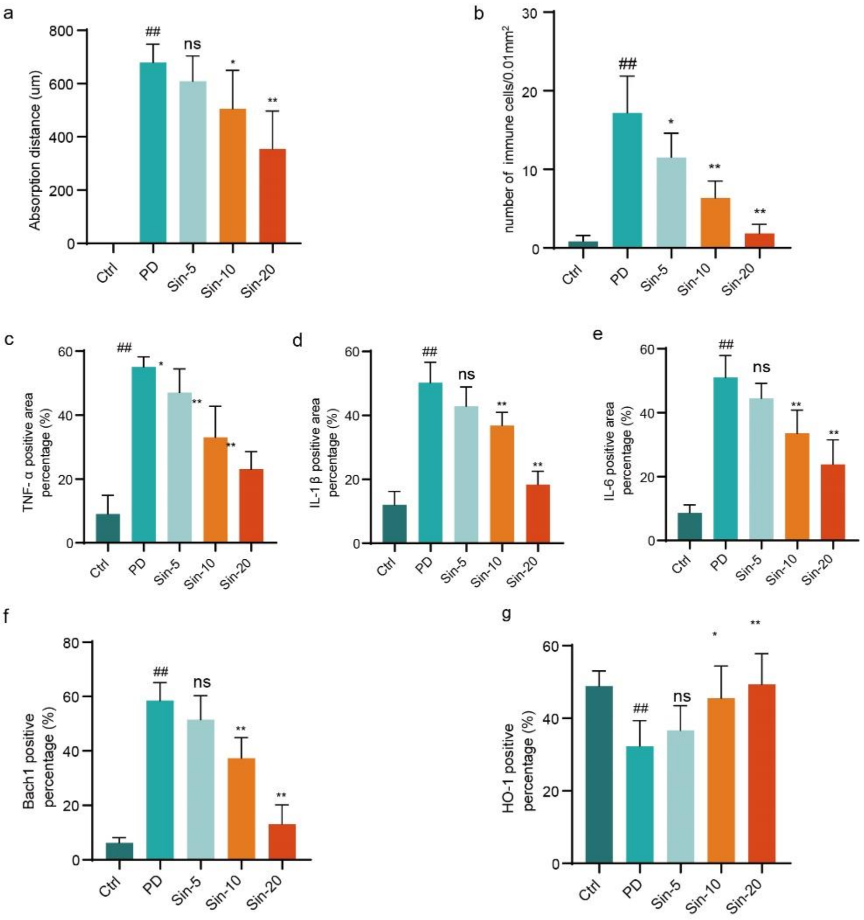

Supplement: Supplementary file 1 — Supplementary Figures [file 41368_2024_305_MOESM1_ESM.pdf]
